# Food availability leads to more connected contact networks among peridomestic zoonotic reservoir hosts

**DOI:** 10.1098/rsos.230809

**Published:** 2023-11-15

**Authors:** Amy J. Kuenzi, Angela D. Luis

**Affiliations:** ^1^ Department of Biology, Montana Technological University, 1300 Park Street, Butte, MT 59701, USA; ^2^ Department of Ecosystem and Conservation Sciences, University of Montana, Missoula, MT 59812, USA

**Keywords:** social network analysis, supplemental feeding, resource provisioning, *Peromyscus maniculatus*, disease transmission

## Abstract

The North American deermouse (*Peromyscus maniculatus*) is a reservoir host for many zoonotic pathogens. Deermice have been well studied, but few studies have attempted to understand social interactions within the species despite these interactions being key to understanding disease transmission. We performed an experiment to determine if supplemental food or nesting material affected social interactions of deermice and tested if interactions increased with increasing population density. We constructed three simulated buildings that received one of three treatments: food, nesting material, or control. Mice were tagged with passive integrated transponder (PIT) tags, and their movement in and out of buildings was monitored with PIT tag readers. PIT tag readings were used to create contact networks, assuming a contact if two deermice were in the same building at the same time. We found that buildings with food led to contact networks that were approximately 10 times more connected than buildings with nesting material or control buildings. We also saw a significant effect of population density on the average number of contacts per individual. These results suggest that food supplementation which is common in peridomestic settings, can significantly increase contacts between reservoir hosts, potentially leading to increased transmission of zoonotic viruses within the reservoir host and from reservoir hosts to humans.

## 1. Introduction

Fundamental to infectious disease ecology is an understanding of how pathogens are maintained and transmitted within populations. With directly transmitted diseases, pathogens are spread via close contact among individuals [1], and factors such as host density, social behaviour, community diversity and resource availability can all play a role in influencing contacts [2–7]. Although contact is a key component in transmission, it is difficult to determine in free-ranging wildlife [8,9].

Researchers have used a variety of methods in attempt to estimate the frequency of contacts between individuals, including visual observation [10], radio telemetry [11], global positioning system (GPS) telemetry collars [12], video camera collars [13] and electronic proximity loggers [14]. While these technologies have proved useful in studying contacts and disease transmission in large mammals such as deer and elk, they are problematic for identifying contacts in smaller, nocturnal mammals such as rodents.

Rodents are important reservoir hosts for many zoonotic pathogens including bacteria (e.g. *Borrelia burdgoferri*, *Yersinia pestis*, *Bartonella* spp.) and viruses (e.g. poxviruses, arenavirus, hantaviruses) [15,16]. The North American deermouse (*Peromyscus maniculatus*) is the most common and widespread mammal in North America and the primary reservoir of Sin Nombre hantavirus (SNV) [17,18]. SNV can spillover to humans causing hantavirus cardio-pulmonary syndrome (HCPS), a serious respiratory illness with a case fatality rate of 35% [19]. Epidemiological studies indicate that hantaviruses (genus Orthohantavirus, family Hantaviridae), including SNV, are primarily transmitted to humans through inhalation or aerosols generated from urine, faeces and/or saliva shed by infected rodents [20–22].

Since the discovery of SNV in 1993, the ecology of the SNV–deermouse system has been well studied in natural environments (see [23] for a review). Longitudinal studies indicate that SNV prevalence in deermice populations varies both spatially and temporally [24–26] and is density dependent [24,27,28]. Many studies have found a male bias in antibody prevalence which suggests that aggressive encounters between males are likely the primary mode of SNV transmission in the wild [23]. However, few studies have attempted to understand SNV transmission in the wild by examining interactions between individuals. Clay *et al*. [29] used fluorescent powder marking to directly monitor contacts and passive integrated transponder (PIT) tags in conjunction with foraging arenas to approximate contacts in deermice in the Utah desert. They found that deermice followed the 20/80 rule [30] with 20% of the individuals in the population being responsible for approximately 80% of the contacts observed [29]. Dizney & Dearing [31] expanded on Clay's work by supplementing PIT tag readers with infrared cameras to examine deermice behaviour at these foraging arenas in Utah. Mice were categorized as bold or shy based on the behaviors observed. Bold mice were less common than shy mice but were more likely to be infected with SNV further suggesting that behavioural heterogeneity may influence SNV transmission in natural environments.

Humans are not generally exposed to SNV in natural environments. Most human cases of HCPS are acquired in peridomestic environments, which include human dwellings and associated out-buildings such as garages, sheds and barns [21,32,33]. Peridomestic environments provide a unique interface between human and deermice populations, but surprisingly few SNV–deermice studies have been conducted in these environments. The few short term studies that have been done have found SNV prevalence of peridomestic deermice populations to be nearly twice as high as populations in more natural environments [26,34,35], indicating that social interactions may differ between natural and peridomestic environments. Thus, it is important to examine these interactions and the factors that may influence them in peridomestic environments.

One way to examine social interactions is through social-network analysis [36]. This type of modelling produces a contact network, a visual representation of individuals (nodes) and their contacts with other individuals (lines or edges) in the population [37]. Social-network analysis is used to quantify social interactions and allows for the characterization of contact heterogeneity which subsequently can be used to infer disease dynamics [37,38]. An individual's degree, or number of contacts, can predict an individual's risk of infection. At the population level, the degree distribution and connectance, or the proportion of all possible connections that are realized, can explain variation in epidemic dynamics [39,40].

One factor that may affect contact between individuals is the availability of resources. Peridomestic settings may provide an environment for deermice where resources such as food and nesting material are more concentrated and accessible compared to more natural environments [41]. The presence of these resources could affect the behaviour of mice [42], leading to increased contacts and subsequently increased transmission of SNV. Another factor that may influence contacts is the population density. There is evidence for density-dependent transmission in this system, implying that contacts increase as population density increases [27]. Deermice populations fluctuate widely both seasonally and annually so examining how population density affects contacts is also important for understanding disease transmission in peridomestic settings and subsequently human risk of exposure to SNV.

Here, we investigate deermouse contact behaviour using a novel approach of simulated buildings, PIT tag technology, and social network analysis in a semi-wild peridomestic setting. We examined if resource provisioning, specifically food and nesting material, and population density affected contact networks. We predicted that individuals would have a higher number of contacts (degree), and the network would be more connected as a whole (connectance), in the presence of added resources and with increasing population density.

## 2. Methods

### 2.1. Deermouse sampling

Our study site was located on a private cattle ranch located near Gregson (Silver Bow County), Montana. Vegetation at the site was predominately big sagebrush (*Artemisia tridentata*) and bitterbrush (*Purshia tridentata*) with scattered willows (*Salix* spp.) and Douglas-fir (*Pseudotsuga menziesii*). The study was conducted from June to October 2002 and from May to December 2003.

Deermice were live-trapped on a 1-hectare trapping grid containing 100 trap stations, with trap stations located 10 m apart. We used standard trapping and handling techniques approved by the American Society of Mammalogists [43], the US Department of Health and Human Services [44], and the IACUC at the University of Montana (050-22AKMTECH-111022). We trapped for three consecutive nights on a bi-monthly basis in 2002 and May–August 2003. From September to December 2003, mice were trapped for three consecutive nights once a month, as logistics related to autumn and winter weather prohibited bi-monthly trapping. Trapping and tagging were conducted immediately prior to the experiments described below, in order to ensure that the majority of mice entering buildings were tagged. At each trap station, we placed a non-folding aluminium Sherman live trap (8 × 9 × 23 cm^3^; H.B. Sherman Trap Co.) containing polyester bedding and baited with peanut butter and oatmeal. Traps were open each evening and checked the following morning. Traps containing animals were transported to a central location for processing. All captured mammals were identified to species. Body mass, sex and reproductive condition (males: testes scrotal or abdominal; females: non-perforate, perforate, pregnant and/or lactating), presence of scars, and location of capture were also recorded. Blood samples were collected from each mouse as described by Kuenzi *et al*. [26] and were tested for SNV antibody as described by Schountz *et al*. [45]. Deermice were ear-tagged with model 1005-1 tags (National Band and Tag Co., Newport, KY). After ear tagging, individuals were also marked with a 12 mm, 134.2 kHz PIT tag (Biomark, Inc., Meridian, ID). PIT tags were injected subcutaneously between the scapulae using a sterile 12-gauge needle. We used a hand-held reader to verify that PIT tags were functioning after implantation, recorded PIT-tag numbers and released each animal at the point of capture.

### 2.2. Simulated building observations

Three simulated buildings (A, B and C) designed to mimic typical outbuildings, such as sheds, that may attract mice, were located in the centre of the trapping grid, approximately 10 m apart. The buildings were small (1.25 × 4.5 × 1.25 m^3^) structures made of wood with a wooden door on one end to allow human access to the building and one mouse sized (3.8 cm diameter circular) opening along the middle of one side of the building. A PIT antenna connected to a data logger (Model 2001F, Biomark Inc., Boise, Idaho) was placed around the mouse-sized opening. PIT-tagged individuals that entered/exited the buildings were detected by the antenna, and time and date of the entrance/exit were recorded. During experiments, we monitored movement into and out of these buildings. Experiments were initiated the night after trapping concluded to minimize the possibility of untagged individuals entering the buildings. In addition, our recapture rates on the last night of trapping were high (ranging from 82 to 95%) indicating that we had tagged the majority of mice in the population. At the start of an experimental run, each building was randomly assigned to either contain food (approx. 1 lb of commercial sweet grain), contain nesting material (approx. 0.5 lb of polyester batting), or remain empty (control). These treatments were switched between buildings approximately every 2 days to keep mice from acclimating to what was inside. For example, for experiment 10, Building A had food, Building B was the control, and Building C had nesting material for 3 days; for experiment 11, Building A was the control, Building B had nesting material, and Building C had food for the next 3 days, and so on. Buildings were opened each evening shortly before sunset and data loggers were turned on. Buildings were closed each morning to limit access to diurnal rodents, and data from the loggers were downloaded. The nesting material treatment did not begin until a month into the experiments, so there are fewer replicates overall for this treatment.

### 2.3. Data analysis and cleaning

All analyses were performed in R v. 4.1.2 [46], and we used the ‘tidyverse’ packages for organizing data [47]. We first examined raw data of the number of PIT tag readings per day per building and treatment. Additionally, because the number of readings could be related to the current density of deermice on the 1-ha trapping grid, we used minimum number alive (MNA) as an index for relative population density. We showed previously that MNA is a relatively unbiased proxy for our system [48]. We performed linear mixed effects regression using the ‘lme4’ [49] and ‘lmerTest’ packages [50], with the response variable, the number of readings per day, logged for normality. The full model included building treatment, MNA, and their interaction as fixed effects, as well as year and building ID as random effects. The ‘lmerTest’ package uses Satterthwaite's method for approximating degrees of freedom [51] for the *t* and *F* tests to calculate *p* values and AIC values [50]. We used the ‘step’ function in the ‘lmerTest’ package to perform automated stepwise elimination of non-significant effects (those with highest *p* value), starting with the random effects and then the fixed effects to find the most parsimonious model, and then corroborated by AIC values.

To create contact networks from these data, we assumed that if two individuals were in the small buildings at the same time, that constituted a ‘contact’. We inferred which individuals were in the building by marking each reading as ‘in’ or ‘out’ based on their sequential order. The first reading per night for each individual was marked as ‘in’ and the next for that individual as ‘out’ and so on. Readings within 15 s of each other were automatically removed by the reader because of potential interference. If a mouse went in and out within 15 s, it would have recorded the ‘in’ and not the ‘out’, which would throw off later readings. To try to correct for this we strategically removed readings. In the uncleaned data, 94% of the durations were shorter than 30 min. We used this as a guide. If there was a time interval that was longer than 30 min, we tried removing the ‘in’ reading that it was associated with, and seeing if that improved the next intervals. We repeated this process until as many readings as possible were less than 30 min. After this correction, 98.5% of readings were under 30 min. Any durations of more than 1 hour were removed from the data set, since durations that are too long will have a disproportionate effect on the contacts networks because it will inflate contacts. We used linear mixed effects regression to examine if the durations spent in buildings were correlated to building treatment (fixed effect) or building ID or year (random effects).

### 2.4. Creation of contact networks

We created several versions of contact networks to address different questions, using the ‘igraph’ package [52] in R. For all networks, a node was an individual mouse, and an edge (or connection) existed between nodes if those two mice ever had a contact. If 2 mice were in the same small building at the same time, we considered that a contact. First, for an overall visualization, we created one contact network for each treatment which shows the summed contacts between individuals in buildings with that treatment over the whole 2-year study. For this visualization, the nesting material treatment network is not directly comparable to the food treatment and control, since there were fewer replicates of this treatment, but we present it for illustrative purposes.

For each network or graph, we calculated basic network statistics. The connectance is the proportion of all possible connections which are present, calculated as *E*/[*N*(*N* − 1)/2], where *E* is the number of edges (connections) and *N* is the number of nodes (individuals); this assumes that contacts are not directed (occur in both directions) and cannot occur with oneself. Degree is the number of individuals each individual is connected to, and mean degree is the mean across all individuals in a network.

Next, we created separate graphs for each experiment–treatment combination. For example, one graph was created for Building A during experiment 1, which contained food for the 3 days of the experiment; this was the food graph for experiment 1. A separate graph was created for experiment 1, Building B, which was the control, and so on. This led to multiple replicates of each treatment (a food graph, a nesting material graph, and a control graph for each approx. 2-day experiment). This allowed us to evaluate if differences in network metrics between treatments were statistically significant using linear regression, while also accounting for how surrounding deermouse density varied over the course of the study. Here, because each experiment–treatment combination is a replicate, we can compare all three treatments directly even though there were fewer replicates for the nesting material treatment. We ran linear mixed effects models where the full model included building treatment, MNA, and their interaction as fixed effects, as well as year and building ID as random effects. The response variables here were mean degree (the average number of contacts per mouse per experiment) and connectance (the proportion of possible contacts that occurred per experiment). We found the most parsimonious models using the ‘step’ function in the ‘lmerTest’ package [50].

Additionally, we examined the importance of individual characteristics on centrality (an individual mouse's importance in the network) by creating one large network that had all the connections over the 2-year study. This allowed us to examine the overall degree distribution. We found a skewed distribution, meaning that a minority of individuals had a majority of the contacts. Therefore, we tested for differences in an individual's degree between males and females and between SNV infected and uninfected individuals, by performing negative binomial generalized linear models (GLMs) using the ‘glm.nb’ function in the ‘MASS' package in R [53].

For all analyses, model assumptions were verified by plotting residuals versus fitted values using the ‘performance’ package in R [54]. Model validation indicated no problems.

## 3. Results

**Figure 1.**
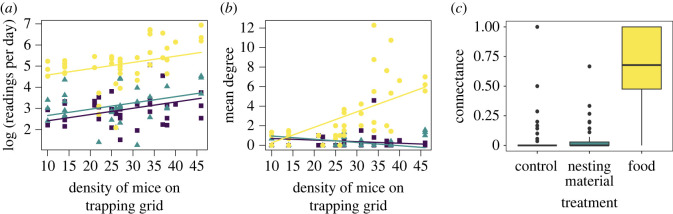
Food supplementation (yellow) had a significant effect on (*a*) number of PIT tag readings per day (logged for normality), (*b*) average number of contacts per individual per experiment (mean degree), and (*c*) connectance (proportion of all possible connections which are present). Deermouse density on the 1-ha trapping grid was also significant for readings per day and mean degree (*x*-axis, *a* and *b*). The food treatment is represented by yellow circles, the nesting material treatment by blue triangles, and the control by purple squares.

The experiments took place over two years for a total of 117 days. Eighty-one unique deermice entered the buildings and had a total of 27 746 PIT tag readings. Deermouse density on the surrounding trapping grid (MNA) varied over time and was higher in year one than in year two (electronic supplementary material, figure S1). Activity patterns of mice (rate of PIT tag readings) were relatively uniform from sunset to sunrise (electronic supplementary material, figure S2). First, we examined determinants of the number of raw PIT tag readings per day, logged for normality. The most parsimonious model included treatment and deermouse density on the surrounding trapping grid (MNA) (*F*_3,108_ = 61.25, Adj *R*^2^ = 0.62, *p* < 2 × 10^16^; electronic supplementary material, tables S1, S2). The number of PIT tag readings per day increased with deermouse density and was significantly higher in the buildings supplemented with food compared to the nesting material treatment and control buildings (figure 1*a*; electronic supplementary material, table S2).

Next, we cleaned the PIT tag detection data as described in the Methods. After data cleaning, 2% of the readings were removed. The durations mice spent in buildings did not statistically differ between treatments (*F*_2,12873_ = 1.87, *p* = 0.154), but there was support for random effects of building ID and year (with slightly longer durations in year 1; electronic supplementary material, tables S3 and S4).

### 3.1. Networks by treatment

**Figure 2.**
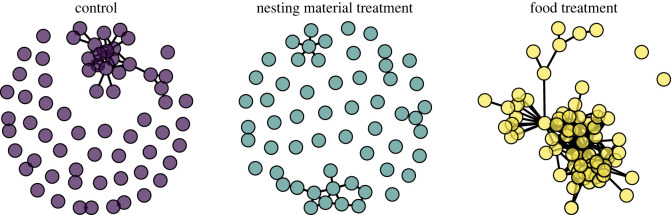
Visualizations of the contact networks for the three different treatments. Nodes (circles) represent individual mice, and edges (lines) represent contact between 2 mice, defined as simultaneous occupancy of small buildings.

First, we created one network per treatment over the whole study to visualize the overall connectivity between the food treatment, the nesting material treatment, and the control (figure 2). Over the 2-year study, the connectance (the percentage of all possible connections that were present) of the food network was 15.3% compared to 2.4% for the control network. The mean degree, or average number of connections per individual, was 10.5 for the food network compared to 1.6 for the control network. Since the nesting material treatment had fewer replicates, its metrics are not directly comparable, but its connectance was 1.6% and mean degree was 0.9.

Next, we created a graph for each experiment–treatment combination, i.e. where each graph spans the time in which a building had a particular treatment (from 1 to 4 days). This led to 42 replicates of the food treatment graph, 32 replicates of the nesting material treatment graph, and 38 replicates of the control graph (table 1). Although treatments did not have the same number of replicates, the metrics for these analyses can be directly compared.

**Table 1.** Summary of graph statistics by experiment–treatment combination. Each graph is a replicate of an approximately 3-day period in which a building had a particular treatment. Shown are the mean of the replicates (and standard deviation).

treatment	no. replicates	nodes	edges	mean degree	connectance
control	38	4.5 (3.0)	1.4 (6.3)	0.3 (0.8)	0.066 (0.185)
nesting material	32	4.7 (3.4)	0.9 (1.8)	0.3 (0.5)	0.063 (0.141)
food	42	5.2 (4.0)	13.1^a^ (21.4)	2.9^a^ (3.0)	0.634^b^ (0.343)

^a^Food treatment is significantly higher including an interaction with current deermouse density.

^b^Food treatment is significantly higher than control or nesting material treatment.

The most parsimonious model for the number of nodes (mice) per experiment included density of mice on the grid (MNA) as a fixed effect (*t* = 2.34, *p* = 0.021) and building ID and year as random effects (Cond *R*^2^ = 0.56; electronic supplementary material, tables S5 and S6). The number of mice entering a building per experiment (nodes) was not statistically different between treatment groups (*F*_2,105.1_ = 1.17, *p* = 0.314; table 1; electronic supplementary material, table S5). However, the number of nodes increased with the density of mice on the grid (MNA), and certain buildings appeared to be in more favourable locations on the grid (electronic supplementary material, table S6). Additionally, more mice entered buildings in year 1.

The most parsimonious models for both the number of edges (connections) and for mean degree (number of contacts per individual) included treatment, deermouse density, and their interaction, as well as a random effect of year (edges: *F*_2,105.1_ = 12.283, *p* < 0.0001, Cond *R*^2^ = 0.44; mean degree: *F*_2,105.1_ = 20.674, *p* < 0.0001, Cond *R*^2^ = 0.60; electronic supplementary material, tables S7 and S9). The most parsimonious model for connectance included only treatment (*F*_2,109_ = 69.37, Adj *R*^2^ = 0.55, *p* < 2 × 10^16^; electronic supplementary material, tables S11, S12). The number of edges, mean degree, and connectance were all significantly higher for the food treatment (table 1, figure 1; electronic supplementary material, tables S8, S10, S12). Additionally, higher deermouse density led to more connections (edges) and higher mean degree but only for the food treatment (figure 1; electronic supplementary material, tables S8 and S10).

### 3.2. Individual centrality

When examining one network over the entire study, we found that the overall degree distribution was skewed, where the top 20% of individuals had 50% of the contacts. There was not a significant difference in degree between males and females by negative binomial GLM (*z* = −1.51, *p* = 0.13). Of the 81 unique mice that entered buildings, 7 were positive for SNV antibody (8.6% seroprevalence), which is an average seroprevalence compared with other Montana sites [25]. There was no significant difference in their degree (*z* = −0.24, *p* = 0.81).

## 4. Discussion

Contact networks are a useful tool for understanding disease transmission and dynamics. However, contacts are difficult to determine in small animals like deermice and other rodents. We used the novel application of PIT technology and experimental buildings to estimate contacts and develop contact networks. This allowed us to demonstrate that food supplementation in peridomestic settings can significantly increase contacts between reservoir hosts, which could lead to increased transmission of zoonotic viruses within the reservoir host and from reservoir hosts to humans. We did not see an effect of supplemental nesting material.

The contact networks for each of the treatments contained roughly the same number of nodes—meaning the number of individual mice that entered each building was not significantly different among treatments. This is not unexpected since all the buildings were available to all the same mice at the same time, and mice have routinely been found to enter non-rodent proofed buildings [55,56]. It also indicates that buildings with resource provisioning were not attracting mice from farther away. However, the number of times a mouse entered a building with food, and thus the number of potential contacts, was significantly higher than a building with nesting material or an empty control building. More entries per mouse equated to more times individuals were in the buildings at the same time, here considered a contact. This increase in contacts led to the food treatment networks being on average 10 times more connected than the control or nesting material networks. Connectance is one of the key determinants of how fast an epidemic spreads across a network [40], often having a greater impact than pathogen characteristics such as infectious period and transmission probability [57].

For our experiment, we artificially added resources to our treatment buildings, which is not uncommon in peridomestic settings. Bags of grain, dog and cat food are often stored in garages, barns or sheds where they may be available to rodents who enter. Spare clothes and bedding are often left in garages or seasonal cabins where they may be used by rodents as nesting material. Other studies have found that anthropogenic food promotes aggregations of wildlife due to being unevenly distributed across the landscape. Food supplementation can increase home range overlap, decrease home range size, increase group size, and reduce territoriality, all affecting contact rates between individuals [58–62]. Of 29 studies that examined how food supplementation affected transmission of a directly transmitted disease, Murray *et al*. [63] found that 95% saw an increase in transmission with supplementation. All of 10 studies that measured contact rates in response to food supplementation found positive relationships [63]. Food supplementation also often increases survival and reproduction [63,64], which can increase the susceptible population and infectious period. However, food supplementation occasionally has positive impacts by increasing the health of individuals and strengthening their immune response to parasites, but it depends on food quality [64–67]. Our experiments were too short to see these effects on population dynamics.

Supplementation with nesting material did not have a significant effect on movement in and out of buildings or on contacts. Our simulated buildings were checked daily and mice were never found to be living in the buildings. These building were not structurally complex and likely did not have favourable thermodynamics to support permanent residence of mice that has been observed in some peridomestic settings [26]. Nesting material may be more important in buildings that support residency.

We found that host density was an important driver of contacts using this method. The experimental buildings were in the middle of a 1-ha trapping grid, and we monitored the density of deermice on this trapping grid monthly. Deermouse population density varied over time (electronic supplementary material, figure S1), and was a significant driver of the raw number of PIT tag readings (the number of times a building was entered or exited; figure 1*a*) and the average number of contacts per individual (mean degree) during an experiment (figure 1*b*). This suggests that deermouse density is important in contact rates between individuals. Previously, the role for density in driving transmission of SNV had been unclear. This issue has mainly been examined by comparing deermouse density to SNV prevalence because measuring contact rates directly is difficult. Previous field studies have found mixed relationships between deermouse density and SNV prevalence, only rarely showing a concurrent positive relationship [68], and often showing either no relationship [68–71], or even a negative relationship [25,72]. However, recent modelling studies using long-term datasets have supported density-dependent transmission—the concept that as density increases, contacts and transmission increase—in this system [27,28]. These studies clarified how temporal variations in host densities can lead to delays between density and prevalence. Few studies have attempted to measure contacts between deermice; however, Clay *et al*. [71] found a positive relationship between probability of intraspecific encounters and deermouse density. Here, we provide additional support for increased deermouse density leading to increased intraspecific contacts.

We saw significant individual heterogeneity in contacts. Woolhouse *et al*. [30] proposed the 20/80 rule whereby 20% of the hosts are responsible for 80% of the transmission of infectious diseases. Multiple studies have supported the general concept that a minority of hosts are responsible for a majority of transmission in many host–pathogen systems, including vector-borne [73], sexually transmitted [74] and directly transmitted [75] disease systems. For deermice, Clay *et al*. [29] found that 20% of the deermice in their study were responsible for 77% of the contacts (using PIT tag readers at foraging arenas). In this study, we saw that 20% of the mice were responsible for 50% of the contacts. While the percentages may differ, this study supports that a minority of individuals are responsible for the majority of contacts and potentially transmission in host–pathogen systems.

We did not see a significant difference in contacts between males and females; however, male deermice often have higher prevalence of SNV [24]. Intraspecific aggressive behaviour, such as biting, is thought to be an important mechanism of transmission of SNV [23]. We were only able to evaluate contact in terms of proximity, but the nature of contact, aggressive or otherwise, would also be important in disease transmission. Aggressive behaviour may be more common in male deermice than females, leading to increased transmission even when spatial proximity is similar. Clay *et al*. also did not see differences in deermouse contact rates between sexes [71]. Future studies incorporating video cameras along with PIT technology in the buildings would aid in evaluating the nature of contact.

Contact networks represent possible transmission pathways through the population. How contacts are defined is key for inference of disease spread. Studies on other wildlife systems have inferred contacts for network analysis by behavioural observation [76–78], radiotracking [79,80] or proximity loggers [14,81]. However, these methods are currently not feasible for small nocturnal rodents. Multiple studies on rodents have assumed contacts using capture–mark–recapture methods if trapped at or near the same location (at the spatial scale of the home range), up to one month later [4,38,82–84]. Although this time frame may be appropriate for parasites such as helminths with external infective stages (e.g. [82,85]), it is not the appropriate time scale for transmission of directly transmitted diseases or those with limited environmental persistence. Here, we considered being in the same small building at the same time an appropriate temporal and spatial scale to define a contact relevant for SNV transmission, since it is thought to be transmitted via bites and grooming and possibly indirectly through inhalation of virus shed in faeces, urine and saliva [23,86].

It is likely that not every mouse in the population was tagged, and some untagged individuals entered buildings. We tried to minimize the number of untagged mice by trapping for 3 consecutive nights prior to the start of our experiments. Our recapture rates on the last night of trapping ranged from 82 to 95% indicating that most, but not all, individuals in the population were tagged. Untagged mice entering buildings would mean that we are missing nodes in the network. Missing nodes can affect inferences of the rate and pathways of spread of disease across a network [87]. However, it is unlikely that untagged individuals show differences in behaviour that makes them more likely to enter buildings of only a certain treatment; therefore our conclusion that food provisioning increases contacts should be unaffected by missing contacts with untagged individuals.

In humans, the risk of contracting rodent-borne diseases is linked to increased probability of exposure to rodents. Research following the 1993 outbreak of HCPS in the Four Corners region of the southwestern United States found higher numbers of mice trapped inside case-patient households compared to control households [32], indicating that human risk of infection increases with the number of mice entering buildings. Similar results have been found for other rodent-borne disease systems. For example, in West Africa, Lassa fever is linked to environmental factors such as poor housing conditions and improper food storage that exposes humans to virus shed by infected *Mastomys* rodents in and around human dwellings [88]. Our finding that food provisioning increases the number of contacts within buildings contributes to the growing body of evidence [89–91] that intervention methods such as rodent proofing and proper food storage (for both reducing exposure to rodents and avoiding food contamination) are important for reducing the risk of rodent-borne diseases.

## 5. Conclusion

Using a novel application of social network analysis, we showed that food supplementation, similar to what would occur in peridomestic settings such as barns, led to an increase in contacts among individual deermice. Given that deermice are the primary reservoir of SNV, a directly transmitted virus, this increase in contacts could lead to increased transmission of virus and may help explain the higher SNV prevalence of infection that has been documented in peridomestic deermice populations. We also demonstrated increased deermouse density leading to increased intraspecific contacts and individual heterogeneity in regards to contacts. Future work should focus on investigating the nature of contacts between individuals as well as the probability of transmission given contact.

## Data Availability

All data and code are included in the electronic supplementary material [92].
